# Atlantic bluefin tuna tagged off Norway show extensive annual migrations, high site-fidelity and dynamic behaviour in the Atlantic Ocean and Mediterranean Sea

**DOI:** 10.1098/rspb.2024.1501

**Published:** 2024-10-09

**Authors:** Keno Ferter, Camille M. L. S. Pagniello, Barbara A. Block, Otte Bjelland, Michael R. Castleton, Sean R. Tracey, Theodore E. J. Reimer, Andreas Sundelöf, Iñigo Onandia, Martin Wiech, Francisco Alemany, Leif Nøttestad

**Affiliations:** ^1^ Institute of Marine Research, Bergen 5005, Norway; ^2^ Hopkins Marine Station, Stanford University, Pacific Grove, CA 93950, USA; ^3^ Hawaiʻi Institute of Marine Biology, University of Hawaiʻi at Mānoa, Kaneohe, HI 96744, USA; ^4^ Institute for Marine and Antarctic Studies, University of Tasmania, Hobart, Tasmania 7001, Australia; ^5^ Department of Aquatic Resources, Institute of Marine Research, Swedish University of Agricultural Sciences, Lysekil 54330, Sweden; ^6^ AZTI Marine Research, Basque Research and Technology Alliance (BRTA), Sukarrieta 48395, Spain; ^7^ ICCAT, International Commission for the Conservation of Atlantic Tunas, Madrid 28002, Spain

**Keywords:** Atlantic bluefin tuna, electronic tagging, *Thunnus thynnus*, annual migration, diving behaviour, foraging

## Abstract

Atlantic bluefin tuna (ABFT; *Thunnus thynnus*) is a highly migratory species. To investigate the migrations and vertical behaviours of ABFT migrating to Nordic waters, we deployed pop-up satellite archival transmitting tags on 25 ABFT off Norway (curved fork length: 228–292 cm). We obtained 16 full-year migrations, which differed between individuals, and physically recovered 13 tags, which provided 4699 days of archival depth and temperature data. ABFT occupied waters from the Arctic Circle to as far south as Cabo Verde, Africa, and occupied depths down to 1190 m and temperatures from 0.5 to 27.8°C. During their annual migrations, ABFT spent, on average, 68 days in Norwegian waters, 65 days in the Newfoundland Basin, 35 days around the Canary Islands and 33 days in the West European Basin. Most ABFT entered the Mediterranean Sea with a mean entry date of 13 May and visited known spawning grounds, staying, on average, 44 days. All ABFT with full-year deployments returned to Norwegian waters. ABFT displayed high site-fidelity and dynamic vertical diving behaviours that varied between hotspots and seasons. These spatiotemporal data provide important ecological knowledge for sustainable management and the conservation of the recently recovered eastern ABFT stock.

## 1. Introduction

Atlantic bluefin tuna (ABFT; *Thunnus thynnus*) is a large, endothermic and highly migratory species [1], which occupies a wide range of habitats in the North Atlantic Ocean and adjacent seas [2]. ABFT is managed as a mixed stock (i.e. western and eastern) based on a management strategy evaluation (MSE) approach adopted by the International Commission for the Conservation of Atlantic Tunas (ICCAT) in 2022 [3]. The western ABFT stock spawns in the Gulf of Mexico, while the eastern stock’s principal spawning grounds are in the Mediterranean Sea [4,5]. Recently, an additional spawning area has been discovered in the Slope Sea off the coast of the United States [6,7], but the genetic origin of these fish remains unresolved.

ABFT are commercially important fish that were overexploited in the last century [8]. While highly abundant up to the middle of the last century, ABFT declined in productive feeding areas in the northeast Atlantic for several decades, corresponding with a steep decline in the size of the eastern ABFT stock [9]. To counteract this major decline, ICCAT implemented a rebuilding programme initiated in 2007 [10]. The objective of the recovery plan was to maintain ABFT at levels that would support maximum sustainable yield. Furthermore, ICCAT agreed by consensus to move to an MSE-based management procedure in 2022, with specific management objectives including status, yield and stability for both stocks [3]. A series of very successful recruitments and strict management measures resulted in the protection of strong eastern-origin year classes (e.g. 2003) and have contributed to an unprecedented increase in stock size and spatial distribution of eastern ABFT in recent years [3]. This, in turn, has led to the reappearance of ABFT in their traditional feeding areas in the northeast Atlantic during summer and autumn [11–14].

ABFT have been observed in Norwegian waters for thousands of years [15]. Being an important fishery resource, ICCAT and Norwegian fisheries logs have recorded the commercial catch of *ca* 270 000 individuals in Norway since 1950 [16] (electronic supplementary material, figure S1). Historically, ABFT arrived in Norwegian waters around July, to feed on highly abundant small planktivorous schooling fish species such as mackerel (*Scombrus scombrus*) and herring (*Clupea harengus*), before they departed again in late autumn [17]. After over a half-century of decline, ABFT have returned to the northeast Atlantic in increasing numbers from 2012 onwards [11]. This increase may have profound effects on predator–prey interactions as ABFT are now targeting schooling fish species [11,18,19]. Conventional tagging studies in the 1950s demonstrated that ABFT tagged in Norway were recaptured in Spain and far north in Norwegian waters up to 1 year later [17]. ABFT tagged in the western Atlantic have also been recaptured in Norwegian waters [20]. However, any detailed knowledge on the full-year migration cycle and vertical diving behaviour of ABFT during their annual migration to their northern distribution limit has been lacking. The resurgence of ABFT in Norwegian waters has provided an opportunity to resolve this knowledge gap by using electronic tagging.

Rapid advances in electronic tagging have made it possible to investigate the horizontal migration and vertical behaviours of ABFT in more detail [4,5,21]. Pop-up satellite archival transmitting (PSAT) tags, which collect depth, temperature and light data, have been deployed on large fish with high success [22]. After a pre-programmed period, they detach and transmit the recorded data via the Argos satellite network. Physical recovery of the tag provides access to the high-resolution data archive, which makes it possible to estimate spatial migrations with higher accuracy and study the vertical diving behaviours of the fish in finer detail.

While there have been extensive long-term electronic tagging studies in the western Atlantic [4,5], electronic studies in the eastern Atlantic, particularly in the northeast Atlantic, have only been initiated in the last two decades [12–14,23]. ABFT have been tagged off the coast of Ireland from 2003 to 2023, showing extensive migrations through the North Atlantic, but only one of these fish entered Norwegian waters [14]. Data from ABFT tagged in Skagerrak between Sweden and Denmark in 2017 indicated fidelity to the North Sea [13]. However, none of these studies has tagged ABFT in waters north of 60°N, which is at the entrance of the productive Norwegian Sea ecosystem [24].

The objectives of our study were to investigate and quantify the annual migrations and vertical diving behaviours of ABFT feeding at their northernmost distribution limit and to record where these fish went during the spawning season. Importantly for management and conservation purposes, knowledge of the migratory pathways, hotspots of presence, and vertical behaviour provide quantitative information on spatiotemporal distributions critical for understanding the migratory biology of adult eastern-origin ABFT.

## 2. Methods

### (a) Capture and tagging

Twenty-five ABFT (curved forked length (CFL) = 228–292 cm, mean ± s.d.: 257.8 ± 17.6 cm) were tagged in the autumn of 2020 (*n* = 5), 2021 (*n* = 9) and 2022 (*n* = 11) off the west coast of Norway (i.e. from 60°N to 63°N) (table 1). This area is on the continental shelf and is, historically, a known feeding area for ABFT [25]. ABFT were captured with custom heavy rods (i.e. ≥130 lb rating) fitted with conventional reels (i.e. ≥size 80 class) and a 400 lb leader, which was connected to squid spreader bars. Fish were hooked by the terminal squid on a J-hook. Eighteen fish were captured from the Institute of Marine Research’s scientific purpose-built tagging vessel. Seven fish were captured by highly experienced recreational anglers and transferred to the tagging vessel. Once at the boat post-hooking (retrieval time varied from 15 to 100 min, mean ± s.d.: 31 ± 19.7 min), ABFT were guided into the boat using a 108 cm wide, custom-built aluminium ramp. A stainless-steel lip hook was used to carefully pull the fish onto the ramp at the stern of the tagging boat [5,26], and the fish were placed on a 6 cm thick foam mattress covered with a tarpaulin. A saltwater hose was used to irrigate the fish’s gills and a wet towel was placed over the eyes.

**Table 1.** Metadata for all 25 tags deployed off Norway between 2020 and 2022. FL, curved fork length. The pop-up date is the date the pin-burn was initiated.

ID	PSAT ID	tagging date	tagging latitude	tagging longitude	CFL (cm)	pop-up date	pop-up latitude	pop-up longitude	duration (days)	status	pop-up reason
5120082	20P1207	31/08/2020	62.5°	5.5°	252	04/06/2021	45.2°	−20.4°	278	transmitted	pin broke
5120083	20P1161	19/09/2020	61.5°	4.3°	244	19/09/2021	61.5°	4.3°	366	recovered	interval
5120084	20P1200	28/09/2020	61.5°	4.5°	250	28/09/2021	61.5°	4.2°	366	recovered	interval
5120085	20P1160	29/09/2020	61.4°	4.3°	266	02/01/2021	43.7°	−18.7°	96	few transmissions	too deep
5120086	20P1191	29/09/2020	61.4°	4.3°	250	29/09/2021	58.7°	5.3°	369	recovered	interval
5120087	20P2966	30/08/2021	62.5°	5.5°	253	02/09/2021	62.5°	5.5°	4	recovered	mortality
5121086	20P2993	16/09/2021	62.3°	4.5°	242	22/07/2022	52.5°	−14.1°	310	transmitted	tag failure^a^
5121087	20P2985	16/09/2021	62.3°	4.5°	290	16/09/2022	62.3°	3.8°	367	recovered	interval
5121088	20P2988	16/09/2021	62.6°	5.3°	270	16/09/2022	62.6°	3.3°	367	recovered	interval
5121089	20P2968	29/09/2021	62.2°	4.8°	243					never transmitted	
5121090	21P0041	29/09/2021	62.2°	4.8°	292	29/09/2022	63.9°	1.4°	366	transmitted	interval
5121091	21P0055	29/09/2021	62.2°	4.8°	275	29/09/2022	64.0°	6.2°	366	transmitted	interval
5121092	21P0051	30/09/2021	62.4°	5.0°	244	20/04/2022	34.0°	−11.5°	203	transmitted	predation
5121093	21P0049	06/10/2021	60.8°	4.5°	275	06/10/2022	61.2°	2.7°	366	recovered	interval
5122081	21P2007	01/09/2022	62.4°	5.2°	250	01/09/2023	37.8°	−0.7°	366	recovered	interval^b^
5122082	21P2006	05/09/2022	61.1°	4.4°	280					never transmitted	
5122083	21P2005	11/09/2022	62.3°	5.0°	228	11/09/2023	57.9°	6.2°	366	recovered	interval
5122084	21P2010	11/09/2022	62.3°	5.0°	240	11/09/2023	62.6°	5.3°	366	recovered	interval
5122085	21P2016	23/09/2022	61.5°	4.4°	250					never transmitted	
5122086	21P0046	23/09/2022	61.6°	4.4°	283	23/09/2023	62.4°	2.9°	366	recovered	interval
5122087	21P0047	24/09/2022	62.1°	4.9°	248	24/09/2023	36°	14.5°	366	few transmissions	interval^b^
5122088	21P0042	24/09/2022	62.1°	4.9°	237	24/09/2023	62.4°	4.6°	366	recovered	interval
5122089	21P0048	24/09/2022	62.2°	4.8°	251	24/09/2023	63.8°	7.8°	366	recovered	interval
5122090	21P0053	28/09/2022	61.4°	4.4°	254	28/09/2023	61.2°	4.3°	366	recovered	interval
5122091	21P0045	29/09/2022	62.6°	5.3°	277	29/09/2023	35.8°	14.7°	366	transmitted	interval^b^

^a^ Tag detached prematurely as it registered ‘floating’ even though it was still attached to the fish.

^b^ Fish was captured in Mediterranean Sea.

All fish were tagged with a PSAT tag (MiniPAT-348, Wildlife Computers, WA, USA). Each tag was fitted with two custom 15 cm tethers that were inserted into the fish’s dorsal musculature using titanium darts next to the second dorsal fin [26]. Tags were programmed for 365 day deployments, with an archival recording frequency of 5 s and a constant pressure release after a 3 day window of ±2.5 m depth. The tag’s auto-depth release was set to 1700 m. Fish were also tagged with a conventional tag (Hallprint Fish Tags, SA, Australia) close to the second dorsal fin on the same side. Additionally, the CFL of each fish was measured. A fin clip was taken from the pelvic or pectoral fins and stored in >99% ethanol for future genetic analyses.

### (b) Geolocation methods and hotspot identification

Daily positions of tagged ABFT were estimated via a three-step process described in detail in [26]. In summary, light-level longitudes and sea surface temperature (SST)-derived latitudes were used as inputs to a Bayesian state space model (SSM) to generate the most probable tracks for each tag [27–29]. Light-level longitudes were calculated using the Global Position Estimator v. 2 algorithm provided by Wildlife Computers. SST-derived latitudes were computed by matching SSTs recorded by the tag to remotely sensed SSTs [30]. Markov chain Monte Carlo (MCMC) methods were used to fit the SSM to these initial position estimates. This involved running two MCMC chains and saving 20 000 position estimates at each 6 h time step to generate posterior probability distributions. The posterior means of the longitude and latitude estimates were used as the most probable tracks.

The SSM accounts for observation error and standardizes position estimates in time, which were sub-sampled back to a 24 h time step for the analyses. It was adapted from the version described in [28] to include a bathymetry mask to ensure that no position estimates were on land or in waters shallower than those recorded by the tag. To visualize position estimation uncertainty [31], 99% likelihood surfaces were generated from the full posterior distribution of all estimated positions using the R package *adehabitatHR* [32]. The posterior distribution for a position estimate had a median (± median absolute deviation (MAD)) width of 1.8 ± 0.1 degrees of longitude and median height of 2.8 ± 0.4 degrees of latitude. Tracks and time series were reviewed and cropped to remove sections after the pop-up date. Horizontal speed (m s^−1^) was computed between subsequent positions for each tag.

Hotspots of ABFT presence were identified from the total number of daily geolocations within 1° × 1° latitude and longitude bins standardized by the proportion of tags in each bin. Boundaries of these hotspots were drawn to enclose regions spanning greater than 4° × 4° with more than the 95th percentile estimated positions in each 1° × 1° bin but also considered known bathymetric structures, oceanographic features, and political borders. The boundaries of the Newfoundland Basin (NB) and West European Basin (WEB) hotspots were previously defined in [14]. The eastern boundary of the Mediterranean Sea (Med) was extended from [14] to 36°E. Two new hotspots were identified in Nordic waters (NOR) and offshore of the Canary Islands (Canaries). The extent of the hotspot in Nordic waters was limited to the Norwegian Exclusive Economic Zone (EEZ) as defined on MarineRegions.org. The extent of the Canaries hotspot was selected to be between 24° and 35°N, and 21° and 9°W. We have omitted the Coastal Ireland hotspot reported in [14] owing to the high median horizontal speeds in these bins, and grouped geolocations from this region with those located outside of the other five hotspots, where median horizontal speeds were also high. We refer to this new grouping as migratory.

### (c) Habitat envelopes

Pressure data were corrected for pressure drift by applying a third-order polynomial fit to the daily minimum depth and subtracted from the raw depth time series to correct for the recovered PSAT’s pressure sensor drift. Depth and temperature data were extracted from the depth-corrected time series for a 24 h period starting at midnight. Temperature data were bin-averaged and vertically interpolated between the daily minimum and maximum depth onto a regular grid of 1 m resolution to create temperature–depth profiles. A median filter with a 20 m window was used to smooth each profile. The mixed layer depth (MLD; m) was estimated from profiles using the threshold definition from [33] of Δ*T* = 0.2°C greater than the temperature at 10 m depth. The temperature at the MLD was also estimated. Profiles whose minimum depth was less than 10 m or spanned less than 25 m in the water column (i.e. the vertical expanse of water stretching between the surface and bottom of the ocean) were omitted. but included when estimating the SST. SST was calculated as the median temperature in the uppermost 5 m of the water column. Daily positions from the Bayesian SSM were linearly interpolated to match the sampling frequency of the data from recovered PSATs. Depth and temperature data were binned every 2 m and 0.5°C, respectively, to create habitat envelopes in each hotspot and each season. Seasons were separated by month. Autumn includes September to November, winter includes December to February, spring includes March to May and summer includes June to August.

### (d) Dive analysis

Individual dives were identified to analyse the spatial and seasonal variability of vertical diving behaviours of ABFT in each hotspot. Dives were defined to start when a fish exceeded a threshold depth and end when a fish returned above this depth. The threshold depths were calculated every 18 h as the median depth in the top 10 m of the water column for each fish. Prior to calculating the threshold, a median filter with a window length of 1 min and 15 s was applied to the depth time series. This window length was empirically selected to remove high-frequency noise from the depth time series. Each individual dive was required to span at least 10 m in the water column. Daily dive frequency, individual dive duration (h) and maximum descent rate (m s^−1^) were calculated. Daily maximum depth (m), median depth (m) during the day and night, as well as percentage time at mesopelagic depths (i.e. depths greater or equal to 200 m) were also computed. To visualize these dive metrics, the median value within 1° × 1° latitude and longitude bins was taken. Owing to position estimation uncertainty, the bin into which each observation was assigned could be shifted by a median of ±1 bin in the east–west direction and ±2 bins in the north–south direction.

### (e) Statistical analysis

Environmental properties and dive metrics derived from depth and temperature time series had non-Gaussian distributions (Lilliefors test, *p*-values < 0.001). Therefore, Kruskal–Wallis (KW) tests, a non-parametric method, were used to determine differences in environmental properties and dive metrics between hotspots and seasons. Tukey’s honestly significant difference (HSD) procedure was employed for *post hoc* comparisons at a significance level of 5%. Fish with tracks lasting less than 365 days and fish that were caught in Mediterranean tuna farms were omitted from summary statistics reported on residency in hotspots as well as entrance and exit dates. We also excluded data recorded after the capture of fish in the dive analyses. Hotspots/season pairs with five or fewer observations were omitted from statistical tests. Spearman’s rank correlation coefficient (*ρ*) was used to test for correlations between environmental properties and dive metrics as well as between dive metrics. Prior to the correlation, the median value of each environmental property and dive metric within 1° × 1° latitude and longitude bins was taken. Median (*x̃*), MAD and/or *post hoc p*-values are reported unless otherwise stated. All means (*µ*) are reported with s.d. (*σ*). KW *χ* ^2^- and *p*-values are reported in electronic supplementary material tables.

## 3. Results

**Figure 1.**
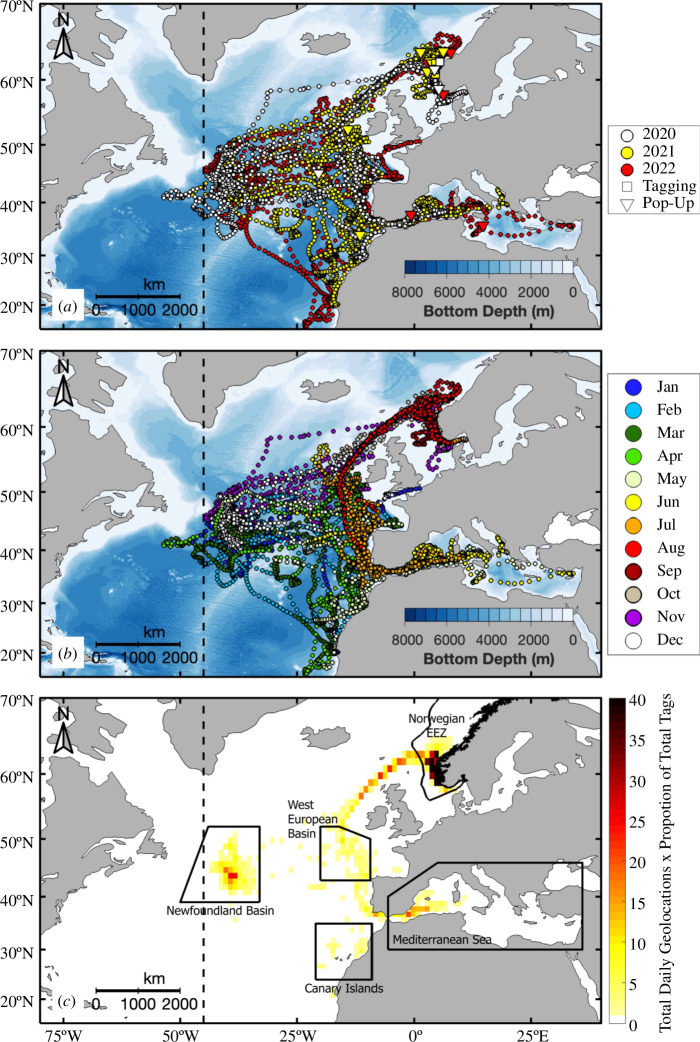
Tracks of electronically tagged Atlantic bluefin tuna (*n* = 19) released off Norway, with daily geolocations (circles) coloured by (*a*) deployment year and (*b*) month. Tagging (square) and pop-up (inverted triangle) positions are also shown in (*a*). (*c*) Number of daily geolocations within 1° × 1° latitude and longitude bins standardized by the proportion of tags in each bin. Boundaries of hotspots are outlined in solid black in (*c*). The ICCAT management line at the 45°W meridian is also shown (dashed black line).

Twenty-two (88%) of the 25 PSATs deployed popped up. Nineteen tags transmitted significant datasets via satellite or were physically recovered (deployment duration: 203–369 days, *µ* ± *σ*: 350 ± 43 days), enabling the generation of most probable tracks (figure 1 and table 1; electronic supplementary material, figures S2–S7). In total, 16 of these 19 tags stayed on the fish for a minimum of 365 days. Thirteen of these full-year tags (76%) were physically recovered, enabling the retrieval and analysis of 4699 days of high-resolution archival depth and temperature data (electronic supplementary material, figure S8 and S9).

**Figure 2.**
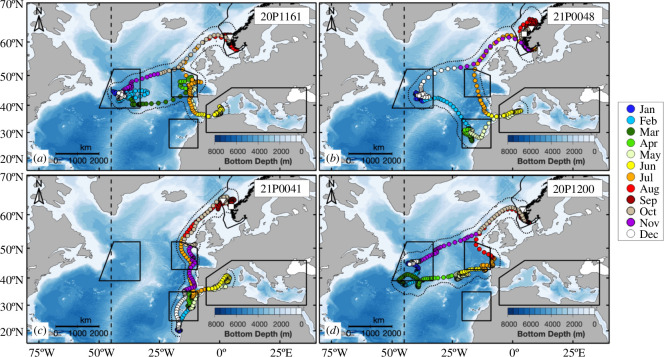
Example tracks of the four different annual migration routes from the present study, with daily geolocations coloured by month. Fish travelled to the (*a*) Newfoundland Basin, West European Basin and Mediterranean Sea, (*b*) Newfoundland Basin, Canaries and Mediterranean Sea, (*c*) Canaries and Mediterranean Sea, and (*d*) Newfoundland Basin, West European Basin and back to the Norwegian Exclusive Economic Zone. Boundaries of hotspots are outlined in solid black. The ICCAT management line at the 45°W meridian (dashed black vertical line) and the extent of the 99% likelihood surface (dotted black line) are also shown. The PSAT number is shown in the top right corner of each panel.

The horizontal migrations of tagged ABFT extended latitudinally from waters off Cabo Verde at 15.4°N to Norwegian waters on the edge of the Arctic Circle at 66.3°N (figure 1*a*). Tuna also moved across a large longitudinal range spanning from 34.0°E in the Mediterranean Sea off Turkey to 53.6°W just south of the Grand Banks in the North Atlantic. Only three individuals crossed ICCAT’s management line, spending 6–39 days west of the 45°W meridian. Hotspots included waters within the Norwegian EEZ, Newfoundland Basin, West European Basin, Canary Islands and Mediterranean Sea (figure 1*c*). ABFT displayed four different annual migration patterns (figure 2).

**Figure 3.**
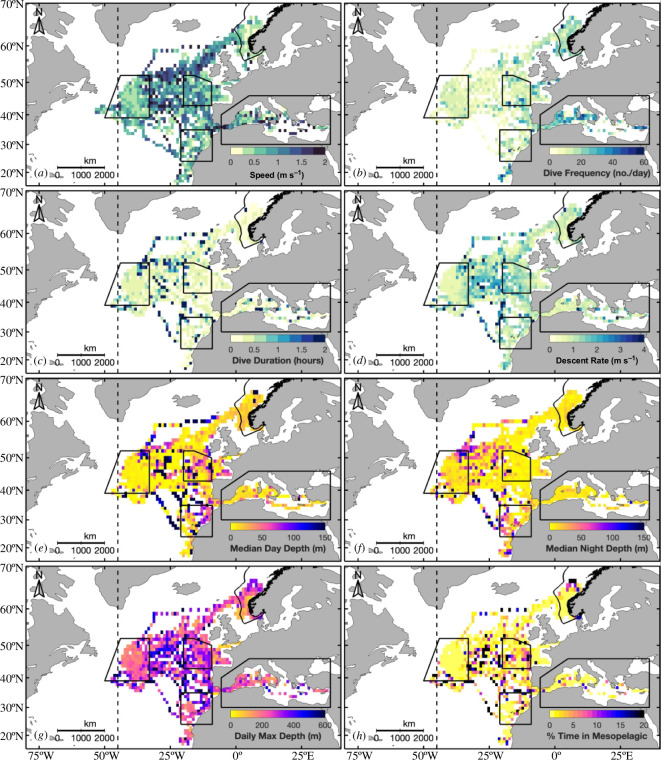
Behaviour of Atlantic bluefin tuna tagged off Norway during their annual migration showing (*a*) horizontal speed (m s^−1^), (*b*) daily dive frequency (number of dives per day), (*c*) dive duration (h), (*d*) maximum descent rate (m s^−1^), (*e*) median day and (*f*) night depth (m), (*g*) daily maximum depth (m), and (*h*) per cent time in mesopelagic (i.e. depths greater than 200 m) within 1°×1° latitude and longitude bins from recovered tags (*n* = 13). Panel (*a*) also includes data from transmitted tags (*n* = 19). Boundaries of hotspots are outlined in solid black. The ICCAT management line at the 45°W meridian is also shown (dashed black line). Summary statistics and *p*-values are reported in electronic supplementary material, tables S10–S18.

**Figure 4.**
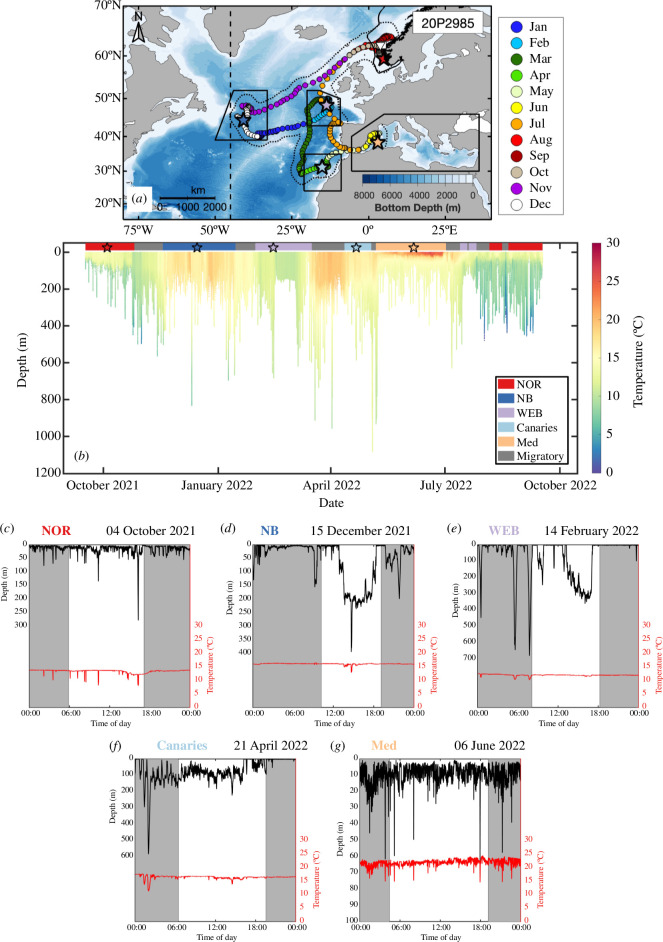
Track and archived depth time series of Atlantic bluefin tuna (ABFT) 20P2985. Daily geolocations in (*a*) are coloured by month. Boundaries of hotspots are outlined in solid black. The ICCAT management line at the 45°W meridian (dashed black vertical line) and the extent of the 99% likelihood surface (dotted black line) are also shown. The depth trace in (*b*) is shaded by archived temperature (°C). Labelled horizontal colour in (*b*) denotes the hotspot in which the ABFT is located (NOR - Norwegian Exclusive Economic Zone, NB - Newfoundland Basin, WEB - West European Basin, Canaries - Canary Islands, Med - Mediterranean Sea). (*c*)–(*g*) Example depth (black) and temperature (red) traces from each hotspot from days indicated with stars in (*a*) and (*b*). Shaded grey areas indicate nighttime.

The vertical diving behaviour of ABFT varied between hotspots (figure 3; electronic supplementary material, figure S10 and tables S10–S18) and seasons (electronic supplementary material, figure S11 and tables S19–S27). Across all hotspots and seasons, ABFT spent most of their time in the top 50 m of the water column (electronic supplementary material, figure S8). The maximum depth recorded was 1190.6 m (electronic supplementary material, figure S5f). Figure 4 shows depth and temperature traces from five different days for one ABFT (20P2985), which is one of three individuals that travelled to all five hotspots. These days were selected as representative examples of the typical vertical diving behaviour in each hotspot, which is described in detail in the following text.

### (a) Departure from Norwegian Exclusive Economic Zone

**Figure 5.**
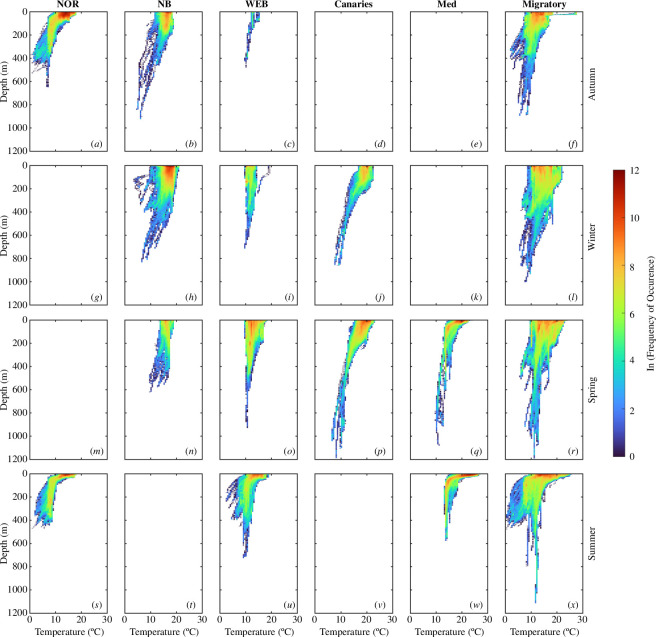
Habitat envelopes by hotspot (NOR - Norwegian Exclusive Economic Zone, NB - Newfoundland Basin, WEB - West European Basin, Canaries - Canary Islands, Med - Mediterranean Sea, and Migratory) and season. Depth and temperature data from recovered PSAT tags (*n* = 13) are binned every 2  m and 0.5°C, respectively. Summary statistics and *p*-values for habitat envelopes are reported in electronic supplementary material, tables S3–S9.

All fish were tagged in the Norwegian EEZ in the autumn, where they spent, on average, 18.4 ± 7.1% of their time (electronic supplementary material, table S1). Median (± MAD) MLD encountered by ABFT in the Norwegian EEZ during the autumn was 26.0 ± 8.7 m and median temperature at the MLD was 13.6 ± 0.9°C (figure 5; electronic supplementary material, table S3). ABFT swam significantly slower in the Norwegian EEZ (0.31 ± 0.18 m s^−1^, *p*-values versus all hotspots <0.001; figure 3*a*; electronic supplementary material, figure S10a and table S10). They also made numerous (17 ± 8 dives per day, *p*-values versus all hotspots <0.001), short duration (0.22 ± 0.15 h, *p*-values versus all hotspots <0.001 except Canaries = 0.79) and shallow (daily maximum depth of 160.0 ± 82.4 m, *p*-values versus all hotspots <0.001) dives (figure 4*c*). Fish departed the Norwegian EEZ throughout the autumn and early winter (7 October to 23 November, *µ* = 23 October), travelling through the coastal waters of Ireland either to the Newfoundland Basin (*n* = 17, CFL *µ* ± *σ*: 255.4 ± 17.8 cm) or towards the Canaries (*n* = 2, CFL *µ* ± *σ*: 281.0 ± 15.6 cm) while swimming against the North Atlantic Current [14].

### (b) Newfoundland Basin

Fish spent, on average, 17.9 ± 9.8% of their time in the Newfoundland Basin (electronic supplementary material, table S1). In this hotspot, deep MLDs and relatively warm temperatures at the MLD were recorded in the autumn to spring (97.2 ± 47.7 m and 16.9 ± 0.7°C across three seasons, *p*-values versus all other hotspots <0.001; figure 5*b,h,n*; electronic supplementary material, table S3). Despite the longest dive durations (0.39 ± 0.28 h, *p*-values versus all hotspots = 0) and lowest daily dive frequency (12 ± 4 dives per day, *p*-values versus all hotspots ≤0.001), ABFT spent little time at mesopelagic depths in the Newfoundland Basin, with a median daily maximum depth of only 201.2 ± 47.8 m. Fish in this hotspot also had the shallowest median depth during the day of any hotspot (5.0 ± 3.1 m, *p*-values versus all hotspots <0.001) and this was the only hotspot whose median depth at night (9.6 ± 6.1 m) was deeper than during the day.

### (c) West European Basin

Eleven ABFT departed the Newfoundland Basin between 15 January and 18 April (*µ* = 23 February), travelling across the Mid-Atlantic Ridge to the West European Basin in the same direction as the North Atlantic Current [14], where they reached peak horizontal speeds (0.73 ± 0.39 m s^−1^, *p*-values versus all hotspots ≤0.05; figure 3*a*; electronic supplementary material, figure S10a and table S10). Median MLDs encountered by ABFT in the West European Basin during the spring were like those in the Newfoundland Basin during all seasons and the Canaries during the winter (78.7 ± 59.2 m, *p*-values versus all other hotspots and seasons <0.001 except NB, all seasons and Canaries, winter; figure 5; electronic supplementary material, table S3). However, temperatures at the MLD were significantly colder (12.3 ± 0.9°C, *p*-values versus all other hotspots and seasons <0.001 except WEB, winter; figure 5*o*; electronic supplementary material, table S3). ABFT spent significantly more time at mesopelagic depths (1.75 ± 1.75%, *p*-values versus all hotspots ≤0.002) and recorded the fastest descent rates (1.1 ± 0.6 m s^−1^, *p*-values versus all hotspots ≤0.05) in the West European Basin. The shallowest median night depths were also recorded in this hotspot (5.0 ± 2.8 m, *p*-values versus all hotspots <0.001).

Of the 11 fish that travelled from Newfoundland to the West European Basin, 7 travelled directly from the West European Basin to the Mediterranean Sea and one headed from the West European Basin to the Canaries before entering the Mediterranean Sea. One fish (20P1207) had its tag pop-up near Antialtair Seamount at the beginning of June, and two (20P1200 and 21P0042), whose CFLs at tagging were 250 and 237 cm, respectively, returned to the Norwegian EEZ from the West European Basin without entering the Mediterranean Sea.

### (d) Canary Islands

The remaining 6 of the 17 ABFT that travelled to the Newfoundland Basin from the Norwegian EEZ departed the Newfoundland Basin early between 18 January and 4 February (*µ* = 29 January), swimming in the same direction as the Azores Current [14], and headed south to the Canaries. Two individuals bypassed the Newfoundland Basin and arrived in the Canaries directly from the Norwegian EEZ on 8 January (20P2988) and 1 December (21P0041), respectively. Median MLD was 29.3 ± 12.4 m and median temperature at the MLD was 19.3 ± 0.8°C in this hotspot during the winter and spring (figure 5*j*,*p*; electronic supplementary material, table S3). Vertical diving behaviour in the Canaries was like that in the West European Basin in terms of daily dive frequencies, dive durations, median day depth and daily maximum depths, which were the deepest of all the hotspots. However, the deepest median night depths were recorded in this hotspot (18.1 ± 14.2 m, *p*-values versus all hotspots ≤0.005). Seven of the eight ABFT remained around the Canaries until, on average, 15 May (range: 5 May to 28 May), when they headed towards the Mediterranean Sea, swimming against the Canary Current [14]. One tag (21P0051) popped-up off the coast of Morocco at the end of April owing to a marine mammal predation on 29 March 2022 as indicated by temperatures up to 36.9°C and no light on the tag sensor.

### (e) Mediterranean Sea

Fifteen ABFT crossed the Strait of Gibraltar and entered the Mediterranean Sea between 4 May and 30 May, with a mean entry date of 13 May (electronic supplementary material, table S2). The CFL of fish upon entry ranged from 233.6 to 293.2 cm, with a mean age of 16.5 ± 3.1 years (based on [34]) (electronic supplementary material, table S2). Three tags popped-up in Mediterranean tuna farm pens (i.e. Malta: *n* = 2; Cartagena, Spain: *n* = 1) after year-long deployments. ABFT that were not captured remained in the Mediterranean Sea for between 32 and 66 days (*µ* ± *σ*: 43.9 ± 20.0 days), with a mean exit date of 2 July (electronic supplementary material, tables S1 and S2). The shallowest (11.8 ± 1.0 m, *p*-values versus all other hotspots and seasons ≤0.002) and warmest (22.2 ± 1.0°C, *p*-values versus all other hotspots and seasons <0.001 except Canaries, winter) MLDs of all hotspots were recorded in the Mediterranean Sea during the summer (figure 5*w*; electronic supplementary material, table S3). Additionally, the highest daily dive frequency (30 ± 10 dives per day, *p*-values versus all hotspots = 0) and shortest dive duration (0.22 ± 0.13 h, *p*-values versus all hotspots <0.001) occurred in the Mediterranean Sea.

### (f) Return to Norwegian Exclusive Economic Zone

After leaving the Mediterranean Sea, the ABFT took a similar migration path back to the Norwegian EEZ through the West European Basin, arriving, on average, on 14 August (range: 24 July–30 August). The mean transit time from the Strait of Gibraltar to the Norwegian EEZ was 40.0 ± 8.7 days despite swimming against ocean currents until they reached the coast of Ireland [14]. When ABFT returned to the Norwegian EEZ in the summer, they encountered significantly shallower MLDs than in the autumn (15.3 ± 2.8 m, *p*-value versus NOR, autumn <0.001), though temperatures at the MLD were similar (13.8 ± 0.6°C, *p*-value versus NOR, autumn = 0.91) (figure 5*a,s*; electronic supplementary material, table S3). All ABFT with full-year deployments returned to Norwegian waters.

### (g) Diving behaviour relative to water column structure

All dive metrics were significantly correlated with the MLD (all *p*-values <0.001 except dive duration *p*-value = 0.05 and descent rate *p*-value = 0.001; electronic supplementary material, figures S12 and S13 and tables S28 and S29). Only the daily dive frequency was negatively correlated (*ρ* = −0.33) with the MLD (i.e. more dives per day when the MLD was shallower). All other dive metrics were positively correlated with the MLD. When the MLD was deeper, the daily maximum depth of ABFT was deeper (*ρ* = 0.14) and fish descended to their maximum depth faster (*ρ* = 0.11). Fish also had longer dive durations (*ρ* = 0.07) and spent more time at mesopelagic depths (*ρ* = 0.18). Additionally, median depths occupied during the day (*ρ* = 0.12) and night (*ρ* = 0.13) were deeper (for day length in each hotspot see electronic supplementary material, figure S14). Only daily dive frequency and the median depth during the day and night were significantly correlated with the temperature at the MLD. Daily dive frequency (*ρ* = 0.22, *p*‐value < 0.001) and median night depth (*ρ* = 0.08, *p*‐value = 0.02) increased with higher temperatures at the MLD while median day depth (*ρ* = −0.07, *p*‐value = 0.04) decreased.

## 4. Discussion

We present the first electronic tagging data for ABFT above 60°N, which is close to the northern edge of the species’ distribution [2,19]. The dataset includes 16 full-year tracks, showing remarkable annual long-distance migrations (i.e. >15 000 km). These new tracks show the extent of the species’ highly migratory movements as the fish move from subtropical areas off Cabo Verde to the Subarctic off Norway. High-resolution archival records from PSATs revealed that ABFT experienced temperatures from 0.5 to 27.8°C (electronic supplementary material, figure S15a) and dived down to 1190 m (electronic supplementary material, figure S15b,c), and that their vertical diving behaviour was related to the oceanographic structure of the water column. Our results provide new ecological knowledge for the sustainable management and conservation of the recently recovered eastern ABFT stock.

### (a) Annual migrations and vertical diving behaviour within feeding areas and spawning grounds

ABFT travelled from the Norwegian EEZ to either (i) the Newfoundland Basin and then to the West European Basin, (ii) the Newfoundland Basin and then to the Canaries, or (iii) the Canaries before entering the Mediterranean Sea. In general, across all hotspots, when MLDs were deeper, fish made fewer, long-duration dives per day, descending to their maximum dive depth faster, reaching deeper daily maximum depths and spending more time at mesopelagic depths. This follows previous studies [14,26,35] and suggests that ABFT expand the amount of vertical space they utilize in the water column as the MLD deepens.

ABFT tagged off the coast of Ireland [12,14] showed similar migratory pathways and hotspots to fish in our study, including the increased use of the Newfoundland Basin as a winter-feeding area in the central Atlantic. However, ABFT in our study spent substantially less time at mesopelagic depths in the Newfoundland Basin hotspot (0.006 ± 0.006%; figure 3*h*; electronic supplementary material, figure S10h and table S10) compared with the fish (3.0 ± 1.3%; [14]) tagged in Ireland. This difference is because only one fish in our study travelled to the southwest corner of the Newfoundland Basin (20P1200; electronic supplementary material, figure S3c,d), where a long-lived, quasi-stationary anticyclonic eddy called the Mann Eddy is located [14]. Here, this ABFT dived considerably deeper (daily maximum depth: 346.7 ± 111.2 m) and spent more time at mesopelagic depths (5.0±4.9%). However, our archival records revealed that ABFT that remained within the bowl-like topography of the Newfoundland Basin made few, long-duration dives to a median daily maximum depth of 201.2 ± 47.8 m (figure 3*g*; electronic supplementary material, figure S10g and table S10), suggesting that ABFT exhibit two distinct diving behaviours within the Newfoundland Basin hotspot. Within the bowl-like topography of the basin, fish are spending increased time in their dives and utilizing the surface scattering layer that extends down to 200 m [36]. Within the Mann Eddy, fish are diving deeper and increasing the amount of time spent at mesopelagic depths, which is consistent with [14] and other recent studies that report increased deep diving behaviour of a variety of top predators within anticyclonic eddies [37].

Fish that travelled to the Newfoundland Basin returned to the eastern Atlantic in the spring (figure 1*b*). Here, they spent time within the West European Basin or in the waters around the Canaries. Some individuals travelled directly from the Norwegian EEZ to the Canaries. ABFT in both the West European Basin and the Canary Islands hotspots had deeper daily maximum depths and spent increased time at mesopelagic depths (figures 3*g,h*; electronic supplementary material, figure S10g,h), consistent with what [14] reported in the West European Basin. Similarities between the vertical diving behaviour in the West European Basin and migratory hotspots are likely driven by the return migration of fish from the Mediterranean Sea back to the Norwegian EEZ during the summer, which goes directly through the West European Basin (figure 1*b*). Thus, the West European Basin serves as both a residency region in the winter/spring and a migratory region in the summer for ABFT. Our findings also align with another study identifying the Canaries as a hotspot for ABFT based on stomach and stable isotope analyses [38]. It is hypothesized that ABFT may be timing their migration with the aggregations of caloric-rich snipefish (*Macroramphosus* sp.), which have been identified in the stomach contents of ABFT in the Canaries [38].

Most ABFT in our study entered the Mediterranean Sea, which is known as the principal spawning ground for the eastern stock [4,5]. ABFT occupied all known spawning areas in the eastern, central and western Mediterranean Sea [2,39], including the Balearic (*n* = 9), Tyrrhenian (*n* = 4), Adriatic (*n* = 1) and Levantine (*n* = 1) seas. Archival data showed that ABFT in the Mediterranean Sea made frequent, shallow, short-duration dives (figure 3; electronic supplementary material, figure S10) and occupied waters with shallow MLDs and median SSTs of 22.8 ± 1.0°C during the summer (electronic supplementary material, table S3). These temperatures are within the range that is considered ideal for ABFT spawning in the Mediterranean Sea [40].

By the end of August, all individuals with full-year tracks not captured in the Mediterranean Sea had returned to the Norwegian EEZ. Several tags popped-up close (i.e. <100 km) to the initial tagging position (table 1). This strong fidelity to foraging grounds has also been described in other studies in the eastern and western Atlantic [7,12–14]. Interestingly, only one of the ABFT tagged off the coast of Ireland swam into Norwegian waters during its annual migration [12,14]. This may be explained by the smaller average size of the Irish-tagged fish (CFL: 215 ± 15 cm) compared with those in our study (CFL: 257.8 ± 17.6 cm; table 1). Size-dependent changes in migratory patterns have been shown for other fish species [41], where larger and older individuals perform longer migrations between spawning and feeding sites. Furthermore, the presence and availability of abundant mackerel feeding on zooplankton in the upper 30 m of the water column above the thermocline during summer and early autumn [18] coincided with the restricted horizontal speeds and shallow diving behaviour documented by our archival data records (figure 3; electronic supplementary material, figure S10). Based on visual observations, catches and stomach samples, mackerel have been an important prey species in Norwegian waters during the last few years [13,18]. This indicates that the high abundance and availability of preferred schooling prey species attract ABFT all the way from the Mediterranean Sea to Subarctic waters. Thus, our study confirms that Norwegian waters are, once again, an important feeding area for ABFT, primarily from August to October [11].

Two fish with year-long tag deployments did not enter the Mediterranean Sea (figure 2*d*) and returned directly to the Norwegian EEZ after visiting the Newfoundland Basin and West European Basin. Such behaviour is similar to the results by [12–14], which also found evidence that some ABFT tagged in the Skagerrak and off Ireland did not occupy the Mediterranean Sea during the spawning season despite their large size. These two fish could have (i) been immature, (ii) skipped spawning if mature (which has been reported for other fish species [42]), or (iii) spawned at an unknown site outside the Mediterranean Sea. However, neither individual spent time in waters whose temperatures were above 22.2 and 18.2°C, respectively, both of which are at the lower end of or below temperatures considered ideal for spawning [40].

Given our tagging success to date, we propose that future studies should attempt longer-duration tag deployments (i.e. >1 year) and utilize additional electronic tag types (e.g. internal geolocation tags or acoustic tags) to gain longer and finer resolution time series that will lead to further insights about the migratory and diving behaviour of ABFT in these waters. Moreover, as there have been rare ABFT sightings as far north as Svalbard, Norway [11], future tagging efforts could aim at tagging fish even further north than in the current study if practically possible.

### (b) Ecological and management implications

After severe international overfishing of ABFT in the last century, the eastern ABFT stock has recovered following strict management measures in the recent decade [3]. Our study demonstrates that ABFT have returned to their historical migration patterns [17] and show high annual fidelity to Nordic waters. Our findings are in line with an increasing number of ABFT observations in the northeast Atlantic during the last decade [11,43] and provide a renewed interest in the species at the northern edge of its range.

The high post-release survival (95%) of ABFT in our study (table 1) is similar to other studies on large ABFT that have used heavy rod-and-line tackle [44]. However, potential sub-lethal effects like relatively higher tailbeat frequency after release have been reported [45]. While the high post-release survival supports the continuation of recreational tagging programmes launched in recent years in the northeast Atlantic, the catch-and-release impacts can be minimized if best practice guidelines are developed and followed for ABFT [45].

All fish in our study were spatially assigned to the eastern ABFT stock (i.e. based on their visitation to the Mediterranean Sea) [5]. Importantly, all but three of these fish did not cross the ICCAT management line at the 45°W meridian, suggesting that large ABFT primarily remain in the eastern Atlantic. Nineteen per cent of the individuals with long-term tracks were captured by purse seines in the Mediterranean Sea and transferred into farm pens. This is a relatively high interception rate. Given that the fish returning to Nordic waters represent the largest, fattest and most fecund individuals of the stock and thus enhance the reproductive output and increase the spawning and recruitment potential of the stock [46], ICCAT should take these new findings into account when managing this stock.

The recurrence of ABFT as a top predator in historical feeding areas can have ecological consequences and should be accounted for in the management of other fish species [47]. ABFT are endothermic fish that require significant caloric intake daily [48] and expend more energy when in cooler seas [49]. Previous studies have shown that if ABFT occurrence increases, schooling fish species can experience higher levels of natural mortality [50]. Severe overfishing of herring [51] and mackerel [52] in the 1960s may have influenced the migration pattern and feeding behaviour of ABFT, resulting in fewer ABFT returning to Nordic waters [25]. During the last decade, however, an unprecedented high biomass consisting of several million tonnes of mackerel has been available for ABFT in the Norwegian Sea during summer and autumn [18]. Managing both the mackerel and the ABFT stock will take new foresight as prey and predator overlap in new and dynamic ways in the northeast Atlantic [47]. Therefore, managers are encouraged to be more conscious about the spatiotemporal movement and behaviour data now available from Atlantic-wide electronic tagging studies such as this one to ensure the continued and long-term sustainable management of the eastern ABFT stock and its prey species.

## Data Availability

All data and codes can be accessed via the Dryad repository [53]. Supplementary material is available online [54].
